# Psychological Outcomes and Quality of Life After Hysterectomy for Benign Diseases: A Prospective Cohort Study

**DOI:** 10.7759/cureus.60871

**Published:** 2024-05-22

**Authors:** Mohamed Ferhi, Nadia Marwen, Ameni Abdeljabbar, Jihenne Mannai

**Affiliations:** 1 Psychiatry, Ibn El Jazzar University Hospital, Kairouan, TUN; 2 Psychiatry, Mohamed Taher Maamouri University Hospital, Nabeul, TUN; 3 Obstetrics and Gynecology, Ibn El Jazzar University Hospital, Kairouan, TUN

**Keywords:** prospective cohort, quality of life, anxiety, depression, psychological impacts, hysterectomy

## Abstract

Background

Hysterectomy is a common surgical procedure performed for benign gynecological diseases. While the physical benefits have been extensively studied, less attention has been given to its impact on psychological well-being and overall quality of life (QoL). This study aimed to assess the psychological outcomes and QoL before and after hysterectomy for benign diseases.

Methodology

This prospective cohort study included women undergoing hysterectomy for benign diseases at Ibn El Jazzar Hospital in Kairouan, Tunisia. The study was conducted from January 2, 2020, to December 31, 2021. We used the Short-Form-36 Health Survey (SF-36) to evaluate the QoL and the Hospital Anxiety and Depression Scale (HADS) to assess psychological outcomes preoperatively and after six months. Data entry and analysis were performed using SPSS version 26 (IBM Corp., Armonk, NY, USA) with the significance level (p) set to 0.05.

Results

Of 84 assessed patients, 60 were included. Following the hysterectomy, there were improvements in QoL and psychological outcomes across all domains, regardless of whether total or subtotal hysterectomy was performed. The mean HADS score for anxiety decreased from 12.57 to 8.77 after hysterectomy and from 14.83 to 9.57 for depression. Moreover, the median SF-36 total score increased from 29.81 to 68.1. We found no statistically significant difference between the two groups in all assessed outcomes.

Conclusions

Hysterectomy for benign conditions, whether total or subtotal, positively impacted symptoms of depression and anxiety, as well as the overall QoL for patients. A thorough preoperative psychiatric assessment is recommended to address and support mental health outcomes in these patients. Future research should consider a larger multicenter approach for a broader application of findings.

## Introduction

Hysterectomy, the surgical removal of the uterus, ranks as the most frequently performed gynecological procedure worldwide [1]. Hysterectomies are classified into two types, namely, those conducted for carcinological diseases and those for benign pathologies, which account for most cases. The benign conditions include uterine fibroids, persistent abnormal uterine bleeding, endometriosis, adenomyosis, and uterine prolapse [2]. Total hysterectomy (TH) involves the removal of the uterus and cervix, whereas subtotal hysterectomy (STH) involves the removal of the uterus while preserving the cervical stump [2].

Hysterectomy is associated with potential complications and risks. Bleeding is a common complication following hysterectomy, regardless of the surgical approach employed [3]. Other potential complications include vesicoureteral injuries, digestive complications, and pelvic static disorders [3]. The psychological impact of hysterectomy on individuals can be significant. Complications with body image after hysterectomy can severely reduce the quality of life (QoL). Individuals may experience an emotional disturbance, including decreased self-esteem and increased self-consciousness, which can lead to withdrawal from social interactions and activities that were once enjoyed. This social isolation can exacerbate feelings of depression and anxiety, further diminishing life satisfaction [4].

While existing literature predominantly addresses the psychological consequences of hysterectomies for malignant reasons, there is a notable gap in research concerning the psychological experiences associated with benign indications. For malignant diseases, patients may exhibit increased anxiety and depression, though these levels are similar to those seen in patients undergoing other gynecological surgeries [5]. Additionally, while hysterectomy can improve mood for some by alleviating distressing symptoms, the benefits may be limited for those with pre-existing psychiatric conditions [6]. Interestingly, studies also indicate that there is no significant psychiatric morbidity after hysterectomy, with partners frequently reporting improved sexual and overall QoL [7,8]. Hence, evaluating the psychological well-being of patients undergoing hysterectomy for benign diseases is crucial, as it can profoundly influence QoL [9]. QoL, increasingly recognized as a significant outcome in medical and nursing research and practice, reflects an individual’s perception of their overall well-being, taking into account their cultural background, values, objectives, and concerns [10]. In this context, our prospective cohort study aimed to assess the psychological impact and QoL in patients undergoing hysterectomy for benign diseases.

## Materials and methods

Study design and eligibility criteria

This study was conducted in the Department of Obstetrics and Gynecology of Ibn El Jazzar University Hospital, Kairouan, Tunisia. Consecutive women between the ages of 40 and 65 who were undergoing hysterectomy for a benign indication from January 2, 2020, to December 31, 2021, were selected for inclusion in this prospective cohort study.

The exclusion criteria were set to ensure minimized confounding factors that may affect the outcomes. Participants were excluded if they had pre-existing psychiatric conditions, including major depressive disorder, anxiety disorders, bipolar disorder, schizophrenia, and sexual disorders diagnosed according to the Diagnostic and Statistical Manual of Mental Disorders, Fifth Edition criteria. The verification of psychiatric conditions was performed through confirmation from treating psychiatrists for patients under psychiatric care. Other exclusion criteria included the need for concomitant surgical interventions such as prolapse repair, hysterectomies performed for hemostatic or carcinological reasons, and incidental discovery of malignancy in the hysterectomy sample postoperatively. Further exclusion criteria included incomplete questionnaire responses, any medical condition that interferes with understanding of the evaluation, and refusal to participate in the study.

Surgical procedures

An initial assessment was performed for all participants to document their medical history and perform a thorough gynecological evaluation. This evaluation included a gynecologic examination, transvaginal ultrasound, and the collection of samples for basic laboratory tests. To standardize perioperative care and minimize variability in treatment outcomes, the same perioperative protocol was implemented across all participating hospitals. This protocol included the administration of perioperative prophylaxis for deep vein thrombosis using low-molecular-weight heparin subcutaneously. Analgesia was the same in all patients. Additionally, to reduce the risk of infection of the surgical site, a single dose of prophylactic antibiotic was administered intravenously during surgery. Deciding on the surgical technique used for hysterectomy was primarily guided by personal preference and the technical expertise of the operating gynecologist. However, the patient’s condition, which determined the indication for hysterectomy, also influenced the choice of the surgical technique. The surgical procedures were discussed with each patient and the suitable hysterectomy type was selected after the patient’s informed consent.

Assessment methods

We collected patient’s sociodemographic and clinical characteristics using medical and obstetrical records. Operating reports and monitoring sheets provided data on the surgical procedures. We assessed anxiety, depression, and QoL both preoperatively and six months postoperatively using two validated questionnaires in the Arabic language [11,12]. The Hospital Anxiety Depression Scale (HADS), a 14-item tool, was utilized for anxiety and depression screening. This self-assessment questionnaire features two sub-scales, each with scores ranging from 0 to 21, culminating in a maximum score of 42. The questionnaire assesses the patient’s condition over the previous week. The scores for each sub-scale are interpreted as follows: from 0 to 7: absence of anxiety and/or depressive disorders; from 8 to 10: doubtful; and from 11 to 21: anxiety and/or depressive disorders. The sum of the two sub-scales is interpreted as follows: from 0 to 14: no anxiety and depressive disorders; from 15 to 42: presence of anxiety and depressive disorders. The HADS depression sub-scale (HADS-D) is widely used for screening depression in the medically ill [13]. The most recent meta-analysis [14] on the accuracy of the HADS-D found that at a cut-off value of 7 or higher, combined sensitivity and specificity were maximized based on 101 studies (82%, 78%), sensitivity and specificity were 74% and 84% for a HADS-D cut-off value of eight or higher and 44% and 95%, respectively, for a cut-off value of 11 or higher. Regarding anxiety screening, for the cut point ≥8 (seven studies), the pooled sensitivity was 0.78 (0.68-0.85) and the pooled specificity was 0.74 [14].

We used the Short Form-36 Health Survey (SF-36) to assess QoL. Developed in 1992, the SF-36 is a reliable instrument for measuring health perception in a general population. It is easy to use, acceptable to patients, and fulfills stringent criteria of reliability and validity [15]. It is one of the most widely used measures of QoL and is considered the gold standard. This scale is used to monitor the health status of populations, assess the impact of morbidity of certain diseases, and evaluate the effects of different therapies [12]. It consists of 36 questions evaluating the last four weeks before the interview. The questions are divided into the following eight dimensions: physical activity, life, and relationships with others, physical pain, perceived health, vitality, limitations due to the psychological state, limitations due to the physical condition, and mental health. Each of the dimensions of the SF-36 is expressed by a score that varies from 0 (most impaired QoL) to 100 (best QoL). The global score corresponds to the sum of the scores for the eight dimensions divided by 8. A global score of less than 30 corresponds to a poor QoL, a score between 30 and 60 corresponds to an average QoL, and a score above 60 corresponds to a good QoL [15].

Ethical considerations

The Ethics Committee of Ibn El Jazzar University Hospital provided official approval for the study to start under approval number 4523. The participants had been informed of the procedure, benefits, nature, follow-up, and right to withdraw at any time without explanation and had given their written consent. Through the coding of all the data and the protection of the acquired data, we guaranteed the confidentiality and anonymity of each woman.

Statistical analysis

Data entry and analysis were performed using the SPSS version 26 (IBM Corp., Armonk, NY, USA). The description of the qualitative variables was done by the observed numbers and frequencies (%). For quantitative variables, the study of the distribution of the data was done by the skewness and kurtosis coefficients and the normality tests. The tests used for assessing normality were the Shapiro-Wilk test, the Kolmogorov-Smirnov test, and the Anderson-Darling test. The description of these variables was done by means and standard deviations in the case of normal distribution. The use of medians and interquartile ranges was necessary in the opposite case. For the analysis of the association between two categorical variables, we used Pearson’s chi-square test for the comparison of percentages. In cases where the conditions for the application of the chi-square test were not validated, Fisher’s test was used. For the analysis of the association between a qualitative variable and a quantitative variable, the Student’s test was used for the comparison of means, and the non-parametric Mann-Whitney test was used in the opposite case. The significance level (p) was 0.05.

## Results

A total of 84 patients were evaluated for eligibility. In total, 13 participants were excluded for various reasons: four due to pre-existing psychiatric conditions, another four due to medication use affecting sexual function, four due to absence of sexual activity in the six months before the study, and one individual declined to participate in the study. Consequently, we included 71 participants for further consideration. However, 11 were subsequently excluded based on further criteria: three were undergoing prolapse repair as a concomitant intervention, three had undergone hysterectomies for hemostasis, one exhibited incidental histological signs of malignancy, and four provided incomplete or no responses to the questionnaire. The final sample size was established at 60 participants, evenly divided into two groups of 30 each: one group undergoing TH and the other undergoing STH.

Table 1 presents the sociodemographic and clinical characteristics of the patients. The mean age was 52.1 ± 7.3 years. All patients were married, with an average marriage duration of 23.6 years. Moreover, 42 (70.0%) patients were in the menopausal period. The most common indications for hysterectomy were uterine leiomyomas in 26 (43.3%) participants, followed by urogenital prolapse and adenomyosis in 12 (20.0%) participants each. Postoperative complications occurred in six (10.0%) cases, with bladder injuries being the most frequent in three (5.0%) cases, followed by surgical-site infection in two (3.3%) participants, and postoperative peritonitis in only one (1.7%) participant.

**Table 1:** Sociodemographic and clinical characteristics of the study participants (n = 60). TH: total hysterectomy; STH: subtotal hysterectomy

Characteristics	Number	Percentage (%)
Age intervals (years)		
40–49	24	40.0
50–59	22	37.0
60–69	14	23.0
Marriage duration (years)		
>20	31	52.0
<20	29	48.0
School level		
Illiterate	15	25.0
Primary	17	28.0
Secondary	16	27.0
Superior	12	20.0
Socioeconomic level		
Bad	27	45.0
Good	33	55.0
Lifestyle habits		
Tobacco	4	6.7
Regular physical activity	5	8.3
Alcohol	0	0.0
Body mass index		
Underweight	1	1.7
Normal	31	51.7
Overweight	11	18.3
Obese	17	28.3
Obstetrical history		
Nulligravid	5	8.3
Nulliparous	5	8.3
History of childbirth	55	91.7
Cesarean section	8	14.5
Vaginal delivery	47	85.5
Instrumental extraction	3	6.4
Postpartum complications	0	0.0
Menopause	42	70.0
Preoperative clinical signs		
Chronic pelvic pain	30	50.0
Abnormal uterine bleeding	18	30.0
Sensation of a ball in the vagina	12	20.0
Surgical indications		
Uterine leiomyomas	26	43.3
Urogenital prolapses	12	20.0
Adenomyosis	12	20.0
Abnormal uterine bleeding	10	16.7
Type of surgery		
Total hysterectomy	30	50.0
Subtotal hysterectomy	30	50.0
TH surgical approach		
Vaginal route	14	46.7
Laparotomy	16	53.3
STH surgical approach		
Laparotomy	30	100.0
Bilateral adnexectomy	43	71.7
Postoperative complications		
Bladder wounds	3	5.0
Postoperative peritonitis	1	1.7
Surgery site infection	2	3.3

Table 2 displays the mean HADS scores, showing a remarkable decrease in anxiety (from 12.6 to 8.8) and depression (from 14.8 to 9.6) after surgery. Additionally, there was a significant increase in median SF-36 domain scores. The median SF-36 total score increased from 29.8 to 68.1.

**Table 2:** Pre and postoperative scores using HADS and SF-36 evaluation. Notes: The p-value for the difference in scores before and after surgery for the total group was highly significant (much less than 0.001). HADS: Hospital Anxiety Depression Scale; HADS-A: the anxiety sub-scale of the Hospital Anxiety Depression Scale; HADS-D: the depression sub-scale of the Hospital Anxiety Depression Scale; SF-36: 36-item Short Form survey

Scales	Preoperative scores, n = 60		Postoperative scores, n = 60		P-value
	Median	Range	Median	Range	
SF-36 (overall)	29.8	20.3–37.4	68.1	42.1–79.7	<0.001
Physical activity	62.5	45.0–70.0	85.0	62.5–100.0	<0.001
Perceived health	40.0	25.0–45.0	50.0	45.0–65.0	<0.001
Physical limitations	0.0	0.0–18.8	75.0	0.0–100.0	<0.001
Psychological limitations	0.0	0.0–0.0	33.3	0.0–100.0	<0.001
Social functioning	37.5	25.0–75.0	75.0	62.5–87.5	<0.001
Pain	35.0	10.0–55.0	65.0	53.1–77.5	<0.001
Vitality	30.0	25.0–45.0	60.0	50.0–73.8	<0.001
Mental health	32.0	24.0–52.0	66.0	53.0–79.0	<0.001
HADS (overall)	27.4 (mean)	18.8–37.0	18.3 (mean)	9.7–26.9	<0.001
HADS-A	12.6 (mean)	8.9–16.3	8.8 (mean)	5.5–12.2	<0.001
HADS-D	14.8 (mean)	9.7–21.0	9.6 (mean)	3.7–15.4	<0.001

Before surgery, 45 (75%) patients had anxiety disorders, and 51 (85%) had depressive disorders. Postoperatively, there was a significant improvement, with a decrease in the frequency of anxiety from 45 (75.0%) to 12 (20.0%) participants and depressive disorders from 51 (85.5%) to 25 (41.7%) participants. The number of patients with a good QoL rating according to the SF-36 increased from 12 (20.0%) to 35 (58.3%), while the number of patients with a poor QoL rating decreased from 31 (51.7%) to 9 (15.0%), as detailed in Table 3.

**Table 3:** HADS and SF-36 evaluation categories. HADS: Hospital Anxiety Depression Scale; HADS-A: the anxiety sub-scale of the Hospital Anxiety Depression Scale; HADS-D: the depression sub-scale of the Hospital Anxiety Depression Scale; SF-36: 36-item Short Form survey; QoL: quality of life

Categories	Preoperative results, n = 60		Postoperative results, n = 60	
	Number	Percentage (%)	Number	Percentage (%)
HADS-A				
Anxiety disorders	45	75.0	12	20.0
Doubtful	11	18.3	24	40.0
No disorders	4	6.7	24	40.0
HADS-D				
Depressive disorders	51	85.0	25	41.7
Doubtful	2	3.3	11	18.3
No disorders	7	11.7	24	40.0
SF-36				
Good QoL	12	20.0	35	58.3
Average QoL	17	28.3	16	26.7
Poor QoL	31	51.7	9	15.0

According to the surgical procedure, the changes in HADS and SF-36 scores between the TH and STH subgroups before and after surgery are presented in Table 4. There was no statistically significant difference between the two groups in terms of anxiety, depression, and QoL scores in all the sub-scales of the HADS and SF-36.

**Table 4:** Comparison of the score changes between the total and the subtotal hysterectomy groups before and after the surgery. HADS: Hospital Anxiety Depression Scale; HADS-A: the anxiety sub-scale of the Hospital Anxiety Depression Scale; HADS-D: the depression sub-scale of the Hospital Anxiety Depression Scale; SF-36: 36-item Short Form survey

Scale	Total hysterectomy		Subtotal hysterectomy		P-value
	Median change	Range	Median change	Range	
SF-36 (overall)	33.2	20.7 to 44.5	41.1	18.2 to 61.8	0.08
Physical activity	30.0	13.7 to 45.0	30.0	12.5 to 41.2	0.68
Perceived health	10.0	5.0 to 25.0	25.0	0.0 to 33.7	0.17
Physical limitations	50.0	0.0 to 100.0	75.0	0.0 to 100.0	0.13
Psychological limitations	0.0	0.0 to 100.0	100.0	0.0 to 100.0	0.25
Social functioning	25.0	6.2 to 50.0	25.0	9.4 to 62.5	0.36
Pain	42.5	7.5 to 55.6	42.5	6.9 to 75.0	0.66
Vitality	35.0	5.0 to 40.0	37.5	16.2 to 45.0	0.32
Mental health	36.0	2.0 to 48.0	36.0	7.0 to 48.0	0.52
HADS (overall)	-13.0 (mean change)	-17.0 to -8.0	-12.5 (mean change)	-18.0 to -4.0	0.93
HADS-A	-6.5 (mean change)	-8.0 to -2.5	-5.5 (mean change)	-8.0 to -0.7	0.81
HADS-D	-8.0 (mean change)	-10.0 to -4.0	-9.0 (mean change)	-11.0 to -2.7	0.53

## Discussion

Our study findings demonstrate a notable improvement in anxiety and depression symptoms, along with a significant increase in median QoL scores. These positive outcomes were consistent across both the TH and STH groups, as evidenced by the comparable scores on the HADS and SF-36 scales.

The high frequency of psychological disorders before surgery can be explained by the intensity of the preoperative symptoms, which, in some cases, can significantly impact daily functioning. Mood disorders are known to precede various surgical interventions, including hysterectomy [16,17]. Specifically, patients often express concerns about the possibility of pelvic neoplasia and attribute their preoperative symptoms to this potential risk. However, it is important to note that the decreased risk of gynecological cancers following hysterectomy can serve as a reassuring factor in addressing these concerns.

It is crucial to recognize that psychiatric disorders may already be present before hysterectomy and may be mistakenly attributed solely to the surgery itself or the underlying pathology that prompted the procedure. Therefore, conducting thorough screening for psychiatric disorders before hysterectomy becomes imperative, especially considering the potential occurrence of mental breakdowns following surgery. Furthermore, numerous studies consistently demonstrate that preoperative psychopathology can predict postoperative psychopathology [18]. While hysterectomy itself does not seem to cause psychopathology, it is worth noting that for women with a psychiatric history, undergoing a hysterectomy can be a tipping point, exacerbating existing psychological challenges. Additionally, previous experiences of abuse may be linked to persistent problems experienced by some women after the procedure [19]. The high incidence of psychological disturbance observed before hysterectomy also underscores the significance of the woman’s partner in providing support. A supportive and empathic partner can help alleviate negative psychological reactions in a woman undergoing a hysterectomy. It is important to recognize that pre-hysterectomy relationship problems often predict further negative developments in the relationship following the surgery. Therefore, both women and men involved in the process require comprehensive information and support both before and after this surgical intervention [20]. Previous studies have challenged the notion that hysterectomy often leads to mood disorders, highlighting that, in most cases, it is associated with a reduction in anxiety and depression following surgery, regardless of the surgical technique employed, including those with or without cervical conservation [21,22].

In addition, a recent meta-analysis of 22 studies [23] with a sample size of 5,978 participants reported a significant decrease in depression and anxiety levels after hysterectomy. It is important to note that hysterectomy may not provide the same benefits to women with pre-existing psychiatric illnesses or those with personality and psychosocial issues [23]. The HADS proved to be particularly valuable in this context. Elevated scores surpassing predefined thresholds should prompt comprehensive psychiatric assessment for accurate diagnosis and optimal treatment planning [24]. Additionally, gathering personal narratives and subjective experiences from patients would be invaluable in identifying potential psychological distress and tailoring support accordingly.

The symbolic significance of the uterus implies that hysterectomy would be perceived as a psychosocial handicap. However, paradoxically, our study revealed a significant improvement in the QoL of patients following hysterectomy. These findings were consistent with the literature, which attributed this improvement to the resolution of the disabling preoperative symptoms which include chronic pelvic pain, menorrhagia, and urinary problems [21,25,26]. It is important to recognize that deeply ingrained ethnic and cultural beliefs persist in many communities, and these factors can have a profound impact on the outcomes of a hysterectomy. Psychological support is strongly recommended before and after surgery to address anxiety and emotional disturbance. Involving patients as much as possible in the decision-making process is essential. Women’s beliefs and expectations concerning the psychological and QoL outcomes should be taken into account to ensure the acceptance of the procedure and treatment adherence.

Our study has several limitations that warrant consideration. The sample size of 60 women may limit the statistical power to detect clinically relevant differences. Furthermore, the absence of randomization introduces the possibility of confounding by baseline disparities in factors affecting psychological outcomes and QoL. Another limitation is the participation of multiple surgeons, all within a single-center setting. This aspect may detract from the external validity of our findings, as the results may not be generalizable across different surgical environments or physician expertise. Future research could benefit from a large multi-center prospective cohort study to improve the generalizability of the findings. Lastly, we acknowledge that the follow-up period did not allow an evaluation of the long-term outcomes. On the other hand, the prospective design of our study allowed for a more robust evaluation by comparing the scores at the two time points. The used scales are validated instruments known for their high specificity and sensitivity. Additionally, these questionnaires were easily accessible to the study population as they were translated into Arabic. Moreover, the HADS and SF-36 scales offer the advantage of reproducibility and sensitivity to changes, making them reliable for assessing the outcomes over time [12,24].

## Conclusions

Our findings found that hysterectomy has a positive impact on the psychological well-being of patients, as evidenced by a significant reduction in anxiety and depression scores following the surgery. Similarly, hysterectomy was associated with improvements in overall QoL. Therefore, it is crucial to conduct thorough screening for underlying psychiatric disorders before performing a hysterectomy. By identifying pre-existing psychiatric conditions, healthcare providers can offer more appropriate support to optimize the mental health outcomes of patients undergoing hysterectomy.
